# Supplementary material for: Glycogen Synthase Kinase-3 Inhibition by CHIR99021 Promotes Alveolar Epithelial Cell Proliferation and Lung Regeneration in the Lipopolysaccharide-Induced Acute Lung Injury Mouse Model
Source: Int J Mol Sci. 2024 Jan 20;25(2):1279. doi: 10.3390/ijms25021279 (PMC10816825; doi:10.3390/ijms25021279)
Supplement: Supplementary file 1 [file ijms-25-01279-s001.zip › ijms-2803903-supplementary.pdf]

Supplementary Figures

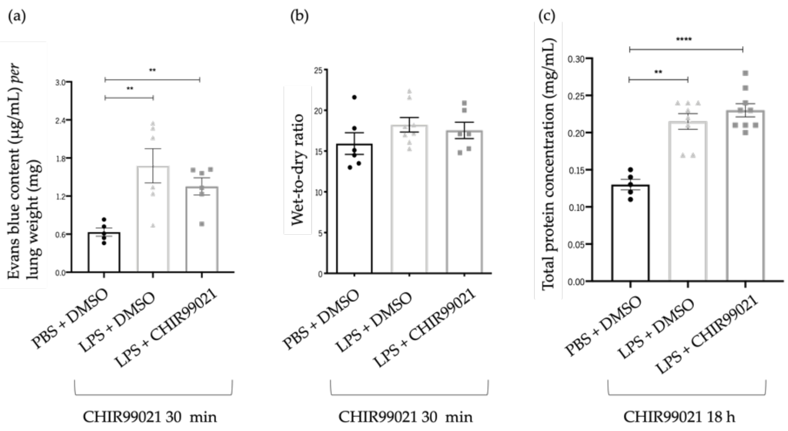

Supplementary Figure 1. Effect of GSK-3 inhibition by CHIR99021 on alveolar-capillary barrier permeability. (a) Quantification of Evans blue dye obtained through a BSA standard curve as µg/mL Evans blue per lung weight (mg) at 24 h after LPS-induced ALI and CHIR99021 treatment at 30 min post-LPS injection. (b) The wet-to-dry lung weight ratio was calculated by the division between the weight of wet lungs and the weight of dry lungs at 24 h after LPS-induced ALI and CHIR99021 treatment at 30 min post-LPS injection. (c) Quantification of total protein content in BALF supernatant as mg/mL at 72 h after LPS-induced ALI and CHIR99021 treatment at 18 h post-LPS injection. (n = 5–9 per group. N=1 independent experiment.). Data are present as mean ± SEM. ** p<0.01, **** p<0.0001, parametric and t-test. BALF: Bronchoalveolar lavage fluid.

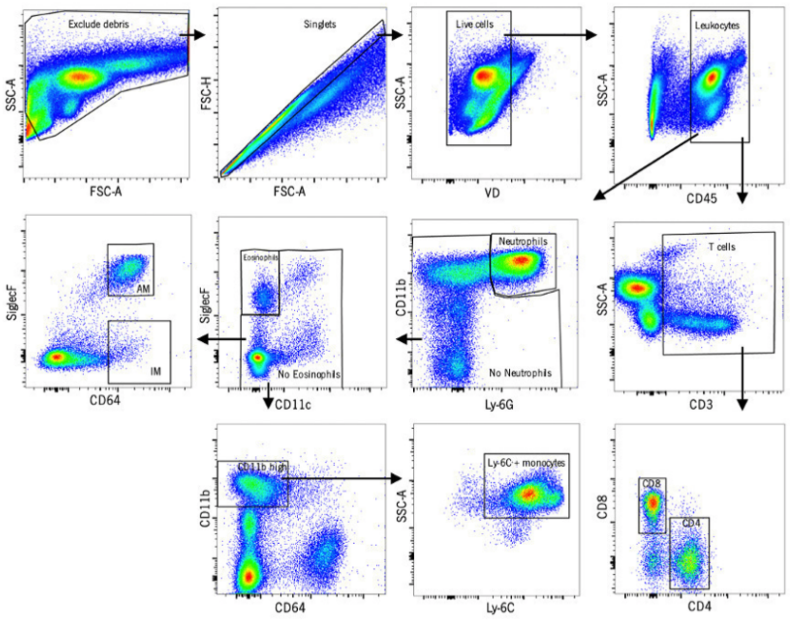

Supplementary Figure 2. The gating strategy used to identify inflammatory cell populations in dissociated mice lungs by flow cytometry. Forward vs side scatter (FSC vs SSC) area and FSC height (H) vs area (A) plots were used for the exclusion of debris and non-single-cell events, respectively. Viable dye (VD) was used to exclude dead cells, and the leukocytes were defined in the $CD45^{+}$ live population (SSC-A vs CD45).

T cells were identified as $CD3^+$ cells (SSC-A vs CD3) within the $CD45^+$ population. $CD8^+$ T cells and $CD4^+$ T cells (CD8 vs CD4) were selected within the $CD3^+$ population. Neutrophils ($CD11b^+Ly\text{-}6G^+$) were also selected within the $CD45^+$ live population. The remaining populations in CD11b vs Ly-6G were considered no neutrophils. Eosinophils ($SiglecF^+CD11c^-$) and no eosinophils were identified within the no neutrophils population. Alveolar macrophages (AM) and interstitial macrophages (IM) were identified within the no eosinophils population as $SiglecF^+CD64^+$ and $SiglecF^-CD64^+$, respectively. $CD11b^+$ high population was also defined within the no eosinophil population (CD11b vs CD64). $Ly\text{-}6C^+$ monocytes (SSC-A vs $Ly\text{-}6C^+$) were identified within the $CD11b^+$ high population.

Supplementary Materials and Methods

Evans Blue dye test

An optimized dose of 200 mg/kg of Evans blue (Sigma-Aldrich, E2129) was intravenously injected through the base of the mouse's tail at 24 h after LPS administration and CHIR99021 treatment at 30 min. After 1 h, animals were euthanized humanely via cervical dislocation, followed by perfusion with 1x PBS. Lungs were then collected, weighed, and dried at 80 ºC for an optimized period of 5 h. Subsequently, the lungs were re-weighed, instantly placed on liquid nitrogen, and macerated. To extract Evans blue, tissue suspensions were incubated with 500 µl of formamide (Sigma-Aldrich, 47671-1L-F) at 60 ºC for 18 h. Next, the samples were centrifuged at 12000 g for 30 min at RT. The concentration of Evans blue in the supernatant was quantified spectrophotometrically using formamide as blank against a bovine serum albumin (BSA) (Sigma-Aldrich, A3294) standard curve ranging from 240 µg/ml to 3.75 µg/ml. Each sample was diluted 1/100 and incubated with Bradford reagent (Sigma, B6916) for 10 min at RT. The absorbance was measured in the microplate reader (Varioskan® Flash-Thermo Fisher Scientific, Vantaa, Finland) at 620 nm. The absorption of Evans blue was corrected for the presence of heme pigments using the formula A_{620} (corrected) = A_{620}-(1.426 x A_{740} + 0.030). Evans blue content was determined as µg/mL Evans blue concentration *per* lung tissue weight (mg).

Wet-to-dry lung weight ratio

The wet-to-dry ratio was calculated by dividing the wet lung's weight by the dry lung's weight. The weights used were obtained as described in the previous section.

Total protein content in bronchoalveolar lavage fluid (BALF)

BALF was collected as detailed in section 4.6. Next, it centrifuged at 1500 rpm for 5 min at 4 ºC. Total protein concentration in BALF was quantified through a standard curve of BSA diluted in 1x PBS, ranging from 0.250 mg/mL to 0.078 mg/mL. 100 µL of the BALF supernatant and curve standard samples were incubated with Bradford reagent (Sigma, B6916) for 10 min at RT and quantified by spectrophotometry at 595 nm in the microplate reader (Varioskan® Flash-Thermo Fisher Scientific, Vantaa, Finland.
